# Mate choice and gene expression signatures associated with nutritional adaptation in the medfly (*Ceratitis capitata*)

**DOI:** 10.1038/s41598-019-42610-2

**Published:** 2019-04-30

**Authors:** Will Nash, Irina Mohorianu, Tracey Chapman

**Affiliations:** 1 School of Biological Sciences, University of East Anglia, Norwich Research Park, Norwich, NR4 7TJ UK; 2 Earlham Institute, Norwich Research Park, Norwich, NR4 7UZ UK; 3 School of Computing Sciences, University of East Anglia, Norwich Research Park, Norwich, NR4 7TJ UK

**Keywords:** Evolution, Genetics

## Abstract

Evolutionary responses to nutrition are key to understanding host shifts and the resulting potential for reproductive isolation. Experimental evolution has previously been used to describe the responses of the medfly (*Ceratitis capitata*) to larval diets with different nutritional properties. Within 30 generations this led to divergence in larval development time, egg to adult survival and adaptation in adult body size. Here we used mRNA-seq to identify differences in gene expression patterns in these same populations, using males from the 60^th^ generation of nutritional selection. We validated differential expression by using qRT-PCR and found that genes linked to metabolism, oxidative phosphorylation and proteolysis were significantly over-represented among the differentially expressed genes. The results provide the first genome-wide survey of the putative mechanisms underpinning evolved responses to nutritional adaptation. In addition, we tested the hypothesis that nutritional adaptation can alter mating patterns. We found evidence for assortative mating by diet at generation 60, but not 90. Hence, the pattern was variable across generations and there was no evidence overall for any isolating mating divergence between the lines. Overall, the results provide insight into the mechanisms underpinning dietary adaptation and extend our knowledge of which traits represent core responses to nutritional selection.

## Introduction

Adaptation to different nutritional ecologies can underpin successful expansion into new habitats and reproductive divergence associated with host shifts with the potential to result in reproductive isolation (RI)^1–3^. For example, in *Rhagoletis* fruit flies^4^ genetic divergence between populations is associated with host plant shifts. If the host shift occurs in sympatry, this can lead to the subsequent formation of host races^5–8^. Similar examples are found in the cactophilic fly *Drosophila mojavensis*^9^ and the pea aphid *Acrythosiphon pisum*^10^. Characteristics of divergent host plants in these herbivorous insects have led to the formation of host races or biotypes, prior to further genetic differentiation between populations^9,11^. Hence, local adaptation and host race formation can lead to divergence in the specific traits that increase fitness on the new host and, via this, potentially result in RI^4,6,12,13^. However, much is still unknown concerning the instigation, and underlying mechanisms, of nutritional adaptation^14–17^. Mechanistic insights can be gained from next generation sequencing (NGS), which is revolutionizing the study of natural selection^18^, adaptation^19,20^ and the initial steps of speciation^21^. NGS methods can also be combined with experimental evolution for additional insights into divergence^22,23^ particularly via ‘evolve & resequence’ techniques^24^. By imposing selection on a particular trait or suites of traits, experimental evolution studies can reveal adaptive responses to selection in real time^25^.

The characterization of patterns of gene expression across genomes offers new opportunities for the study of the putative causal links between differences in gene expression and adaptive genetic divergence^26^. For example, populations of *Drosophila mojavensis* from different regions of the Sonoran desert exhibit premating isolation, mediated by cuticular hydrocarbon profiles (CHCs) and courtship song, based on the cactus host they utilise for reproduction^9,27,28^. Transcriptomic studies have identified a small suite of metabolic, olfactory and behavioural trait genes whose expression is directly influenced by the host food plant^29–31^. The focal species used here, the medfly *Ceratitis capitata*, is an extreme generalist and exhibits wide plasticity in host selection, utilization^32,33^ and oviposition^33,34^. Larvae are viable in a wide range of fruits and diets, from both inside and outside of the known host range^35–38^. This highly plastic host choice behaviour is evident in reports that the medfly can infest over 350 different host fruits^39^ including those of very different nutritional compositions such as citrus fruits, coffee, olives, argan, apples, pears and cherries. As such, the medfly is of economic importance and its ability to exhibit strong nutritional plasticity^36,40^ is thought to have facilitated its global invasion^41,42^. Geographically distinct populations of medfly vary in a wide range of demographic^43–46^ and behavioural traits^47,48^, as well as in genetic structure^41,49^. However, unlike for some Drosophilid species (e.g.^50,51^), there are as yet no reports of mating isolation between medfly strains utilizing different diets^52^.

Here we used mRNA sequencing (mRNA-seq) to identify the responses of protein-coding gene expression to selection under two divergent larval dietary treatments in the medfly (*Ceratitis capitata*), a global agricultural pest. We aimed to capture the genome-wide gene expression signatures associated with the previously-described divergence and nutritional adaptation in these lines^53^. In addition, we extended the range of phenotypes investigated, by testing for divergence in mating preferences. Experimental evolution was conducted on two sets of three independent evolutionary replicates, each selected on different larval diets with different nutritional complexity and caloric value^53^. A previous study showed that within 30 generations, this dietary selection led to divergence in larval development time and egg to adult survival^53^. There was also evidence for the emergence of local adaptation in adult body size, with individuals being significantly heavier when raised on the diet on which they had been selected^53^. In the current study, we tested for evolved differences in gene expression and conducted separate tests for evidence of divergent mating preferences, in these same populations. We used mRNA-seq to characterise the gene expression profiles of pools of sexually mature males from the Starch and ASG lines following 60 generations of nutritional selection. To measure evolved rather than proximate dietary responses we used a standard method of testing individuals that had been reared for 2 generations on a nutritionally intermediate common garden diet. We found evidence for evolved differences in gene expression patterns between the lines and the responses were highly consistent across biological replicates. We identified 214 transcripts as differentially expressed (DE) between the dietary treatments. DE was observed in a suite of genes involved in nutrient metabolism, oxidative phosphorylation (OXPHOS) and proteolysis. These differences may underpin the previously reported divergence in developmental survival and adult body mass. However, mating patterns across generations 60 and 90 were variable, providing no evidence for assortative mating by diet.

## Methods

### Experimental evolution lines

The experimental evolution (EE) lines were derived from the TOLIMAN wild type (originating from Guatemala and reared in the laboratory since 1990^54^). This is an outbred, domesticated strain that harbours selectable genetic variation. For at least two years prior to the start of these experiments the TOLIMAN strain was reared on a wheat bran diet (24% wheat bran, 16% sugar, 8% yeast, 0.6% citric acid, 0.5% sodium benzoate). To initiate the experimental evolution, flies were established on (i) sucrose-based ‘ASG’ medium (1% agar, 7.4% sugar, 6.7% maize, 4.75% yeast, 2.5% Nipagin (10% in ethanol), 0.2% propionic acid, 684 kcal/L) or (ii) ‘Starch’ (S) medium (1.5% agar, 3% starch, 5% yeast, 0.4% propionic acid, 291 kcal/L)^53^. These diets differ in the quality and quantity of both dietary and preservative components and were selected for use as they are known to successfully support larval growth^36^ whilst providing marked differences in both calorific value and carbohydrate complexity. There is as yet little evidence that differences in the presence and concentration of preservatives used will impact on larval life history responses in a manner that could confound our interpretation of nutritional responses^55^.

Three independent biological replicates of each of the two regimes were maintained under allopatry and each was tested in the RNA-seq and mating test experiments. However, within each of these three replicate regime treatments we ran multiple sub replicates (as specified) for the mating tests (otherwise we would test each regime only ‘once’). The qPCR tests were conducted on each of the 3 EE replicates, with technical replication as standard. All experiments and culturing were conducted at 25 °C, 50% relative humidity, on a 12:12 light dark photoperiod. Adults emerging from each replicate were maintained each generation in groups of approximately 30 males and 30 females in plastic cages (9 × 9 × 9 cm). Adults from all lines received the same standard adult diet (*ad libitum* access to sucrose-yeast food; 3:1 w/w sugar:yeast hydrolysate). Each generation, egg density was standardised by placing approximately 500 eggs on 100 ml of the appropriate diet in a glass bottle. When third instar larvae started to ‘jump’ from the larval medium, the bottles were laid on sand and pupae allowed to emerge for seven days. Pupae were then sieved from the sand and held in 9 mm petri dishes until adult eclosion and the propagation of the next generation.

### Identification of gene expression patterns in medfly populations exhibiting divergent nutritional responses, using mRNA-seq

Eggs were derived from the ASG (A) and Starch (S) experimental evolution lines at generation 60 and reared on a common garden glucose (CG) diet (1.5% agar, 3% glucose, 5% yeast, 0.5% propionic acid) under standard conditions and density for two generations. CG was chosen to represent an approximate nutritionally intermediate diet on which larvae from both regimes could be reared through for two generations and placed in an appropriate common garden, to minimise parental effects and at the same time clearly delineate evolved gene expression responses. All adults received the same 3:1 sucrose:yeast hydrolysate diet throughout. The use of a common garden diet is standard to minimise proximate ‘immediate’ responses, in this case to different diets, and hence to accurately measure evolved responses (we took a similar approach below for the mating tests, by using diet ‘swaps’). As the lines originated from the same base population held for ≥2 years on the same diet prior to the initiation of the experiments, the same genetic variation was subjected to the two different diet treatments. The mRNA-seq experiment hence captures evolved gene expression differences that have occurred over time since the start of the experiment. The CG larval diet we used is also known from previous work^36^ to fully support larval development in a manner comparable to that of standard control diets and was hence a suitable diet for this purpose. In this study, we subjected adult males, rather than larvae, to RNA-seq, as we wished to capture the potential effects of divergent nutritional selection on behavioural and reproductive traits related to mating success. To generate the samples for mRNA-seq, adults emerging from daily collected cohorts of pupae were sex-sorted and reared in single sex cages until seven days post eclosion (which is the age of peak sexual maturity in males^56^). Samples of 17–30 male flies formed the pool of mRNA for each replicate of each evolutionary treatment. The flies were flash frozen in liquid N_2_ 30 minutes after lights on (09:30 (GMT)), being the start of the 12 h light period), in groups of 10–15 in Eppendorf tubes and stored at −80 °C until RNA extraction.

### RNA extraction and mRNA-seq

Prior to RNA extraction, males were split into Head/Thorax (HT), and Abdomen (Ab), over dry ice. This allowed us to capture body-part-specific signatures of gene expression and to minimise tissue swamping effects. Total RNA was extracted from samples of 17–22 body parts pooled within each replicate of each dietary treatment, using the *mir*Vana kit (Ambion), according to the manufacturer’s instructions. 5 µg (>200 ng/µl) of Total RNA from male flies was submitted for sequencing. mRNA-seq was conducted (BaseClear provider) on libraries prepared using the Illumina TruSeq protocol, following polyA selection for mRNA. Sequencing was single-end, 50 cycles on the Illumina HiSeq2500 platform (Rapid Mode). Samples were sequenced over 3 lanes (4 samples per lane) using a balanced design, to control for batch effects. Identical sequencing indices were used for each body part and biological replicate from the two different diet regimes, across lanes.

### Bioinformatics

We analysed the RNA-seq data using the bioinformatics pipeline described in^57,58^, which is based upon a subsampling normalisation. This allows good preservation of the original distributions of gene abundances and gene expression following normalisation and hence good sensitivity in detection of subtle gene expression patterns underlying behaviour. Direct comparisons have shown the outputs of this pipeline are fully comparable, in terms of the number and identity of genes showing DE, to established pipelines such as egdeR and DEseq2^57^.

#### Quality control

Initial quality control was performed on original FASTQ files, which were then converted into FASTA format. Reads containing Ns (<1%) were discarded. The set of redundant (R) reads was collapsed into non-redundant (NR) format and the complexity (ratio of NR:R reads) calculated^57,59^. To evaluate the extent of sequencing bias, the per-base nucleotide composition was determined. The proportion of reads incident to the reference genome and available annotations was calculated before and after normalization of expression levels, to ensure that the normalization targeted technical inter-sample variability without altering the biological features present^57^.

#### Read mapping and normalization

The mapping of all reads was done using PaTMaN^60^. This method can be used for aligning short reads (here, our 50 nt mRNA fragments) and was selected because it is a deterministic, rather than probabilistic approach (hence it identifies all hits on the genome/transcriptome). We first evaluated the proportions of genome matching reads resulting from mapping with 0, 1 and 2 mismatches, to balance the number of reads mapped versus accuracy (data not shown). From this, we chose to map with a maximum of 1 mis-match at any position along each read, full length, with no gaps allowed. This yielded a higher proportion of genome matching reads over 0 mismatches and increasing it to 2 gave only a negligible increase in proportion mapping. We used the Ccap1.1 version of the *Ceratitis capitata* genome and curated *C. capitata* transcripts downloaded from NCBI (for rRNAs and protein coding genes). First, rRNA-reads were excluded from all samples and we then sampled the filtered reads to a fixed total of 29 M (the minimum sequencing depth, post filtering), using subsampling without replacement^57^. Gene expression was calculated as the algebraic sum of the abundances of incident reads. Any remaining variability in the expression matrix was then minimised by using quantile normalisation^57,61^. The proportion of reads incident to the mRNA annotations corresponding to v1.1 of the *C. capitata* genome was significantly lower than the proportion of genome matching reads. Hence, we used curated *C. capitata* transcripts downloaded from NCBI^62^, which yielded comparable proportion of transcript- and genome-matching reads.

#### Differential expression analysis

We first investigated differential expression (DE) between replicates, using a log_2_ offset fold change (OFC), with empirically-determined offset of 20^57^ to filter out low level noise. Transcripts with a normalized abundance across all samples of <100 were excluded. The DE analysis was conducted using a hierarchical approach on two levels^58^: (i) body part (tissue) (HT/Ab) and (ii) dietary treatment (A/S). This allowed the calling of DE in transcripts that were body-part-specific and those that were expressed in both tissues. The DE call between treatment types was made on maximal confidence intervals (CIs) and genes with a log_2_(OFC) > 1 (corresponding to a 2-fold change difference) were called as DE.

#### Annotation and functional description

The functional annotation of the current *C. capitata* genome is relatively limited. Hence to utilise our data to gain the strongest inference, whilst still employing a conservative bioinformatic approach, we also used the *D. melanogaster* genome (with its enhanced annotation quality) to strengthen our descriptions of transcript functions. DE transcripts were matched to their corresponding *Drosophila melanogaster* homologues using tBLASTx^63^. The similarity threshold for matching was alignment length > 50 nt, with similarity >50%. The tissue-specific gene lists resulting from this were tested for functional enrichment using g:Profiler^64^ using all *D. melanogaster* genes as the background set. The analysis settings used were g:GOSt with the g:SCS multiple test correction applied^64^.

### qRT-PCR validation

15 candidate genes of interest (GOIs) for validation were chosen, based on their expression levels and to cover representative GO categories and include genes showing both body part-specific and global expression patterns. Presence plots were used to enhance primer design (i.e. to avoid primers spanning exon/exon boundaries). We first assessed the stability of expression of 3 reference genes^65,66^ in our mRNA-seq data. We selected 3 non DE reference genes *(CcRpL13a, CcRpL27*, *poll*) based on a circular permutation test, high read abundance (>500,000 reads in each sample, in both body parts) and by assessing the consistency of expression between tissues (log_2_(DE) < = 0.0001) and treatments. The use of three reference genes gives statistical robustness to reliably infer the reference level of constitutive gene expression in each of the tissues tested. We then designed primers (using primerBLAST^67^) and checked using PatMaN^60^ for potential multiple matches to other genomic locations (primer details in Table S1).

For primer optimisation we used mRNA extracted from whole wild type flies. We removed DNA using TURBO™ DNase (Life Technologies) on 2 μg total RNA. DNase was deactivated using the resin in the TURBO™ DNA-free kit (Life Technologies) and all samples were tested for DNA contamination with no-reverse transcription controls. The QuantiTect Reverse Transcription Kit (Qiagen) was used to reverse transcribe 1 μg total RNA to cDNA, which was then stored at −20 °C. qRT-PCRs were run using a StepOnePlus™ machine (Life Technologies) using iTaq Universal SYBR^®^ Green Supermix with triplicate technical replicates using 10 ng RNA as template in 20 μl reactions in MicroAmp^®^ Fast Optical 96-Well Reaction Plate with Barcode, 0.1 ml (Life Technologies). The qRT-PCR conditions were 95 °C for 30 seconds followed by 40 cycles of 95 °C for 15 seconds and 60 °C for 1 minute and data acquisition. Following the qRT-PCR we ran a melt curve analysis (on default settings). All samples showed a single peak on the melt curve. We included a no template control for each GOI on each plate. All primers were used at a final concentration of 5 μM. For primer optimisation (Eurofins MWG Operon) we produced standard curves using at least five 1:5 dilutions of RNA starting at 50 ng cDNA. qRT-PCR to validate each GOI was conducted on an aliquot of the original total RNA extraction supplied to the sequencing provider for mRNA-seq. Samples where no signal was detected during qRT-PCR were given a nominal CT value of 40, to allow statistical testing. The difference in 2^−**Δ**CT^ between treatments was tested using a two-sample t-test for each GOI.

### Tests for assortative mating by diet

In order to further understand the adaptation reported in our previous study^53^, we extended our phenotypic assays of the selection lines described above to include assays of adult mating preferences. We conducted multiple choice mate choice tests at generation 60 and 90 of selection under the two dietary regimes. Eggs were seeded onto the same common garden glucose (CG) larval diet described above, for two generations prior to testing. This allowed the potential for mating patterns to be linked to the underlying genome-wide expression patterns measured in the mRNA-seq experiment. All mating tests at each generation were conducted simultaneously. At generation 90, we conducted additional tests between replicates within each dietary treatment to test for the contribution of genetic drift. Flies were sorted by sex within 24 hours of eclosion to ensure virginity. Experimental flies were reared in standard 0.8L rearing cages. To enable identification, males and females from one population in each mating test were marked with a spot of red paint on the dorsal side of the thorax. Treatments were fully controlled for handling effects and paint marking.

A multiple-choice design was employed to maximise the opportunity for mate choice. Five days post eclosion and 48 hours prior to the mating tests, 25 A and 25 S regime females and 25 A and S regime males were placed into two, single sex, 0.8L rearing cages. The two cages were connected via a sliding door. Both cages were supplied with 3:1 sugar:yeast hydrolysate diet mix *ad libitum*. Mating tests were conducted when the flies were 7–8 days post eclosion (at peak sexual maturity^56^), with sexes and treatments balanced for age composition. Mating tests were initiated at 09:30, 30 minutes after lights on, by slowly raising the sliding door. Mating pairs were gently removed and placed in numbered 1.5 ml eppendorf tubes for later identification. Three replicates were conducted on each test day, starting at 30 min intervals. The order in which the replicate pairs were tested was alternated, to control for effects of different start times. Each assay continued until 25 mated pairs had been collected, or until 30 minutes had elapsed. The collection of 25 pairs amounted to half of the total population in the cages. Therefore, any effect of diminishing choice due to removal of flies from the cage was minimized^68^. At generation 60, four replicates were conducted for each combination of tests. At generation 90, five replicates each of 16–25 individuals of each sex per line were tested for the own diet and common garden mating tests. For the within line mating tests, four replicates of 25 starting individuals of each sex were tested for the A v A mating assays, and three replicates of 19–25 individuals of each sex per line were tested for S v S. The identity of both individuals in each mating pair was recorded. As only 50% of matings were sampled, mating pairs were treated as independent^68,69^ and results were pooled by line replicate prior to further analysis.

### Statistical analysis

The number of observed and total possible pairings for each pair type was calculated. These raw data were then analysed using JMATING v1.0^70^ to calculate descriptive coefficients based on the cross product estimator of isolation^71^. *I*_PSI_, a joint isolation index, varying from −1 to 1 (with +1 being total assortative mating, −1 total dissasortative mating and 0 random mating^69^) was used to describe total isolation. The PTI coefficient described positive and negative preferences for mating pairs within each line pair, at each time point. An index of mating asymmetry (*IA*_PSI_) captured the difference in frequency between homotypic and heterotypic pairs^72^. Values of *IA*_PSI_ centre around 1 (no asymmetry), with values below one reflecting asymmetry towards the first pair type, and >1 representing asymmetry towards the second. Finally, we calculated *W*, the cross product estimator of sexual selection, to compare the sexual fitness of males and females^70^ and to give the relative fitness of each treatment in comparison to the fittest treatment within each line pairing. The significance of PSI, PSS, and PTI coefficients was determined as the bootstrap probability of rejecting the null hypothesis of random distribution, after 10,000 iterations of resampling^71^. When applied to the isolation index (*I*_PSI_), the asymmetry index (*IA*_PSI_), and the estimator of sexual selection (*W*), this is the two-tail bootstrap probability that the value is significantly different from one (i.e. random mating, or zero asymmetry). To test for the overall effect of diet in RI as indicated by *I*_PSI_, *IA*_PSI_ or *W*, probability values for each line replicate comparison were combined using Fisher’s sum of logs method, implemented with the ‘metap’ package in R^73^. All other data handling and statistical analysis was conducted in R^74^.

## Results

### mRNA-SEQ

#### Quality control

Raw FASTQ files contained between 34.3 M and 53.2 M reads. Following conversion to FASTA format and exclusion of reads containing Ns, we accepted >99% reads for every sample (Table S2). Other initial checks, such as the nucleotide composition across reads, revealed the expected RNA-seq bias, which was consistent for all samples^57^. Next, to optimize the alignment step, the files were transformed from redundant to non-redundant format, yielding between 7.6 M and 10.2 M unique (non-redundant) reads, with a complexity of between 0.192 and 0.23. The tight range of complexities indicated a reliable sequencing output. The variation in complexities observed between replicates, derived primarily from variation in sequencing depth, suggested that a subsampling normalization would be appropriate^57,58^.

#### Genome matching and normalisation of expression levels

First, rRNA mapping reads were excluded. Next, the resulting reads were subsampled at a fixed total (29 M). The non-redundant read count varied between 7 M and 8.2 M reads, with complexity between 0.235 and 0.277, showing that the variation in complexity between replicates was reduced following normalization (Table S2). On the normalized data, the proportion of reads matching to the Ccap1.1 genome was: redundant 80 to 83%, non-redundant 77%. The proportion of redundant reads incident to the genome was in line with the results observed for the original data, suggesting that the normalization successfully minimised technical variability. The proportion of reads matching to *Ceratitis capitata* NCBI transcripts was: redundant 75 to 83%, non-redundant 75 to 79% (Table S2). This was similar to the proportion of genome matching reads, a result that was expected as polyA selection was employed during the sample preparation for the mRNA-seq. As the *C. capitata* NCBI transcript annotations provided a similar proportion of R/NR mapping reads to the reference genome, this dataset was used for the subsequent analysis.

### Identification of differentially expressed protein-coding genes

**Figure 1:**
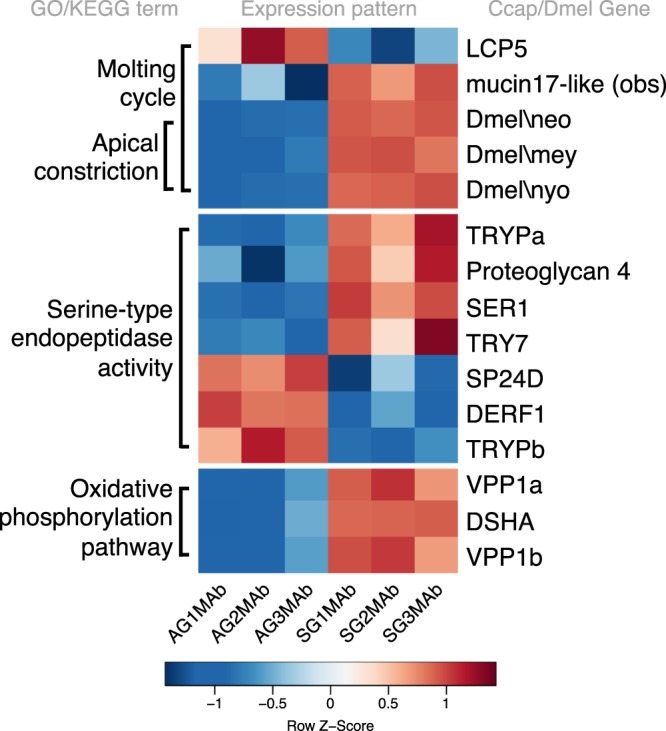
Differentially expressed transcripts in Abdominal tissue of males after 60 generations of adaptation to divergent larval diets. Colour represents row wise Z-score of expression levels normalised by row mean and standard deviation, red represents comparative up regulation, blue represents comparative down regulation. Groupings based on GO enrichment of *D. melanogaster* homologues of medfly DE transcripts are displayed on the left side of the heat map. Gene names are displayed on the right-hand side. ASG samples (AG) are displayed in the 3 left hand columns of the heat map, Starch samples (SG) in the 3 right hand columns, each for replicates 1–3, male abdomen (MAb).

The normalisation provided comparable distributions of expression across samples, with good agreement between replicates throughout (Fig. S1). The amplitude and frequency of DE (Table S3A–D) between tissues was greater than for diets (Fig. S2). A total of 359 transcripts showed DE between treatments (Table 1), with 198 being expressed higher in Starch flies, and 161 higher in ASG. These transcripts were matched to *D. melanogaster* homologues using tBLASTx, which yielded a total of 94 matching genes, 29 expressed in the HT tissue and 65 in Ab. Tests for functional enrichment of this set highlighted 3 enriched GO terms and 1 KEGG pathway, associated with 15 *C. capitata* transcripts (Fig. 1). To test whether unidentified DE transcripts were enriched for transposable elements (TEs), we used BLAST with relaxed parameters (megablast, nr/nt database, not limited by organism, word size: 28 (default)) to check every transcript called as DE that did not bear a *C. capitata* annotation on NCBI, or that was annotated as *C. capitata* unclassified. In the HT there were 30 such transcripts and only one (GAMC01015130.1) returned a BLAST hit related to TEs. In the Ab, of 70 such transcripts, none returned a hit relating to TEs, though GAMC01015130.1 matched to an LTR retrotransposon known only from *Ceratitis*. Overall, unidentified sequences did not enrich for TEs.

**Table 1:** Differentially expressed (DE) transcripts used for functional enrichment.

	Tissue	
	Head/Thorax	Abdomen
**Total DE transcripts**	**122**	**237**
# DE, Starch > ASG	43	151
# DE, ASG > Starch	75	86
*D.melanogaster* homologues	29	65
GO/KEGG terms enriched	0	4

DE between treatment types was called on maximal confidence intervals, with genes exhibiting a log_2_(OFC) > 1 (corresponding to a 2-fold change difference) classed as DE. From this set tBLASTx^63^ was used to retrieve homologues from *Drosophila melanogaster*. These homologues were functionally enriched using g:Profiler^64^. The number of transcripts which were retained in the analysis at each stage, and the resulting number of enrichment terms are displayed per tissue.

#### Differential gene expression in the head + thorax

Of the 122 transcripts that yielded a signature of DE in the HT, 43 were expressed at higher levels in ASG flies, and 75 higher in Starch (Table S3A,B). 29 of these were matched to *D. melanogaster* identifiers following tBLASTx searching. Functional enrichment did not return any significant terms. After removal of duplicates present due to isoform variation, 65 DE transcripts remained (Table S3B). Of these, 22 bore informative NCBI predicted annotations and 9 were predicted as uncharacterised loci. As examples from this set, homologues for the *D. melanogaster* serine protease genes γTry and CG34458, as well as cytochrome P450 genes Cyp6a9 and Cyp6a21 were expressed at higher levels in Starch flies. Consistent with this, several transcripts predicted to be serine proteases also showed higher expression in Starch flies (e.g. XM_004517825.1, XM_004517721.1). Homologues of the developmentally associated larval cuticle protein genes Lcp3 and Lcp56Ab1 were expressed at higher levels in ASG flies. The functional significance of the enrichment for serine proteases in Starch and cuticle genes in ASG flies is not yet clear.

#### Differential gene expression in the abdomen

237 transcripts were DE in abdomen, with 151 expressed higher in Starch flies and 86 higher in ASG (Table S3C,D). Following tBLASTx, 65 *D. melanogaster* identifiers were retrieved. Functional enrichment of these revealed significant overrepresentation for genes involved in the molting cycle (GO-BP:0042303, *P* = 0.0432) and apical constriction (GO:0003383, *P* = 0.0493). Additional enrichment was observed in the molecular function GO term serine-type endopeptidase activity (GO:0004252, *P* = 0.0399) and in genes associated with the oxidative phosphorylation KEGG pathway (ko00190, *P* = 0.0495; Fig. 1). The biological process (BP) ‘molting cycle’ was associated with 5 *C. capitata* genes. Of these, the majority were expressed at a higher level in Starch flies (Fig. 1). However, a single *C. capitata* gene (LCP5) showed the opposite pattern and was expressed at higher levels in ASG flies. This pattern of LCP5 expression was also seen in the HT for ASG flies. Of the five genes enriched for molting cycle, 3 were also enriched for apical constriction. These genes were all expressed at higher levels in Starch than in ASG flies. For all three of these transcripts there were no *C. capitata* annotations, and hence these genes are referred to by their *D. melanogaster* identifiers in Fig. 1. The majority of genes associated with a functional enrichment were serine-like peptidases. 7 *C. capitata* transcripts were associated with this GO term, and exhibited a bidirectional expression pattern, with 4 expressed at higher levels in Starch than in ASG, and the remaining 3 showing the opposite pattern. The final functional enrichment information came from 3 *C. capitata* transcripts that were associated with the KEGG pathway describing oxidative phosphorylation, these were all expressed at higher levels in Starch flies.

After duplicates were removed, 152 abdomen transcripts showing DE remained (Table S3B). Of these, 53 were associated with *C. capitata* annotations or predicted annotations and 21 were predicted as uncharacterised *C. capitata* loci. Within this set were 7 genes with *D. melanogaster* homologues that were functionally enriched for serine peptidase activity (up in ASG: *CG3916, CG32808, Ser6*; up in Starch: *lint, CG34458, γTry, Jon65Aiii*). Further to this enrichment, another *D. melanogaster* gene with a serine endopeptidase domain (CG6800) was DE, with higher expression in ASG abdomens. 4 transcripts with predicted annotations as *C. capitata* serine proteases were also called DE (up in ASG: GAMC01001732.1; up in Starch: XM_004517721.1, XM_004517825.1, XM_012306933.1). 5 *C. capitata* transcripts with *D. melanogaster* homologues showed functional enrichment for the molting cycle (*Cht6, Lcp65Ab1* (*C. capitata* LCP5), *mey, neo, nyo*). Three of these (mey, neo, nyo) also showed enrichment for apical constriction. 3 *C. capitata* transcripts with *D. melanogaster* homologues contributed to the enrichment of the oxidative phosphorylation KEGG pathway (*SdhA, Vha100-2, Vha100-4*).

Beyond the transcripts described by functional enrichment, there was a wide range of additional *C. capitata* transcripts with *D. melanogaster* homologues showing DE. Of particular interest was a transcription factor associated with behaviour in *D. melanogaster* (*acj6*), which is associated with odorant receptor gene expression, as well as another transcription factor (CG15073). Given the dietary selection imposed, it was also interesting to observe DE in *ppk28*, a gene involved in response to water and osmoregulation in *D. melanogaster*^75^ and in Ent2, with reported roles in stress/stress signalling and associative learning in *D. melanogaster*^76^. Predicted annotations of several of the *C. capitata* transcripts lacking *D. melanogaster* homologues was also interesting, as within this set there were one odorant receptor (XM_004534136.1 (LOC101450382, Odorant receptor 67a-like)) and one odorant binding protein (XM_004521128.2 (Obp99a)) and the *C. capitata* gene CYP4E6 (AF028819.1), a cytochrome P450 associated with the processing of extrinsic and intrinsic metabolites^77^.

### Validation by qRT-PCR

Variation in gene expression as determined by mRNA-seq was validated using qRT-PCR (Tables S1 and S4). There was extremely close agreement between qRT-PCR and mRNA-seq expression. The pattern of gene expression was identical in all replicates for all 15 GOIs tested (Figs S3–S17; Table S4) except one (GAMC01008957.1, AG1HT/SG1HT, Fig. S10). Statistical testing showed significant (*P* < 0.05) differences in 2^−**Δ**CT^ values between ASG and Starch flies in 14 out of 20 tests (Table S4). Within the set of validated GOIs, genes matching *D. melanogaster* homologues, such as SER1 (GAMC01003520.1) and TRY7 (GAMC01004081.1) were also validated. Overall, the qRT-PCR experiments provided a strong validation of the bioinformatics analysis of the mRNA-seq data, with 119 out of 120 qRT-PCR tests showing the same direction and/or magnitude as the mRNA-seq.

### Mate choice tests

**Figure 2:**
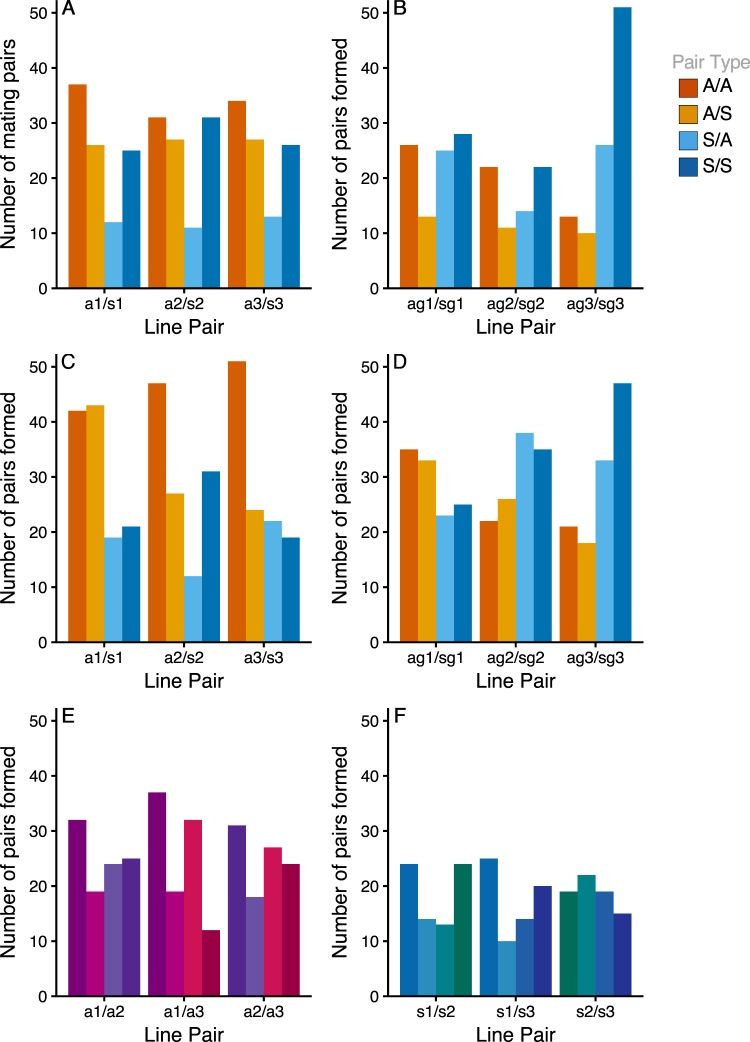
The number of mating pairs formed in multiple choice mating tests between ASG and Starch dietary selection lines after 60 & 90 generations of selection. Each plot shows three replicates, with lowercase letters representing diet (ASG = a or Starch = s) and replicate information (1–3). Upper case letters represent ‘pair types’ formed within that replicate (homotypic = AA, SS; heterotypic = AS, SA). Dark Orange bars represent homotypic pairings between the ASG males and females. Light orange bars represent heterotypic pairings composed of a male from ASG, and a female from Starch. Light blue bars represent the opposite heterotypic pairing, Starch male and ASG female. Dark blue bars represent homotypic matings between a Starch male and female. (**A**) Flies reared on their own larval diet, tested at 60 generations. (**B**) Flies reared on a common garden glucose diet for two generations prior to testing, tested at 60 generations. (**C**) Flies reared on their own larval diet, tested at 90 generations. (**D**) Flies reared on a common garden glucose diet for two generations prior to testing, tested at 90 generations. (**E**) Flies from ASG line replicates tested against other ASG line replicates. Colours represent hetero- and homotypic matings by the same light/dark pattern as above, but with an individual colour pair for each line replicate. All flies reared on their own larval diet, tested at 90 generations. (**F**) Flies from Starch line replicates tested against other Starch line replicates. Colouration as in (**E**). All flies reared on their own larval diet, tested at 90 generations.

At generation 60, there was a predominance of homotypic pairs (i.e. pairing between flies from the same diet regimes, Fig. 2, Table S5) and S males were observed to mate most frequently (Fig. 2, Table S5). However, at generation 90, which also included tests to indicate the presence of genetic drift, these patterns were no longer evident. Hence, overall, there was no evidence of diet-associated mating preferences. The results are described in more detail, below.

#### Sexual isolation (PSI), sexual selection (PSS) and total isolation (PTI) indices

At generation 60, there was a significant deviation from random PTI (Tables S6 and S7) driven by significant deviations from random PSI (Tables S6 and S7). This was evident as homotypic (diet assortative) pairings generally exhibited sexual isolation (PSI) > 1, and heterotypic (diet disassortative) pairings showed negative values. Total isolation (PTI) was significantly >1 for ‘AA’ mating type pairs in 2/3 replicates and significantly <1 for the ‘AB’ mating pair types for all lines (0.44–0.52). In the common garden tests, 2/3 replicates (Tables S6 and S8) showed no significant effect of treatment on PSI and the sexual selection index (PSS) was also not significantly different from one in 2/3 replicates. PTI was significantly <1 for all ‘BA’ mating type replicates (0.4–0.64). Hence at generation 60 there was evidence for significant assortative mating by diet in both the ‘on diet’ and common garden tests. A competitive advantage of ASG males was evident ‘on diet’, mediated by sexual selection. At generation 90, on diet mating tests again showed significant deviations from random PTI (Tables S6 and S9) and a significant deviation from random PSI in a single replicate (Table S9). However, all line replicates showed significant deviations from random PSS (Table S9), indicating that the assortative mating by diet observed at generation 60 was not repeated. This was also found in the common garden tests, where only one line showed significant deviation from random PTI, PSI or PSS (Tables S6 and S10). This effect was not driven by PSI and not linked to assortative mating by diet. At generation 90 the line replicates were also tested against each other to test directly for genetic drift. Within the ASG regime, significant deviation from random PTI was observed in only one replicate (Tables S6 and S11), driven by significant deviation in PSS, again suggesting no assortative mating. There was no significant deviation from PTI in the Starch regime (Table S11).

#### Analysis of isolation - IPSI - Isolation index

At generation 60, all lines showed significant assortative mating when tested on their own (*X*^2^ = 27.03, d.f. = 6, *P* < 0.001) or common garden (*X*^2^ = 19.7, d.f. = 6, *P* = 0.003) diets (Table S12). However, at generation 90 this signature of assortative mating was not observed (Fisher’s Combined test, *X*^2^ = 5.3, d.f. = 6, *P* = 0.51). Tests of lines within dietary regimes showed a significant isolation index in Starch (*X*^2^ = 19.3, d.f. = 6, *P* = 0.004) but not ASG regime (*X*^2^ = 7.5, d.f. = 6, *P* = 0.26) flies.

#### Asymmetry in matings - IAPSI - Asymmetry index

In generation 60, *IA*_PSI_ (i.e. asymmetry in the proportion of homo- versus heterotypic matings) was consistently significantly <1 for heterotypic pairs (‘BA’ mating type) (Fisher’s *X*^2^ = 23.22, d.f. = 6, *P* < 0.001). The frequency of heterotypic pairs was skewed towards BA type matings (i.e. S females and ASG males). No such asymmetry was observed in generation 90.

#### Divergence in W - Cross product estimator of sexual selection

There was no significant difference between treatments in female-specific *W* at generation 60. However, *W* for S males was lower than for ASG in generation 60 (Fisher’s *X*^2^ = 24.28, d.f. = 6, *P* < 0.001) and 90 (*X*^2^ = 15.76, d.f. = 6, *P* = 0.015).

## Discussion

We observed evolved differences in the expression patterns of genes involved in metabolism, development and OXPHOS in adult males from experimentally evolved populations subjected to divergent nutritional selection. These differences may underlie the phenotypic signatures of adaptation in body mass previously described^53^. Independent tests of mating preference in the same populations provided no consistent evidence for assortative mating by diet between the populations at the same generational timepoints. mRNA-seq successfully captured variation in gene expression in sexually mature males from both diet regimes. As all flies were reared on a common garden diet prior to RNA extraction, the measurement of evolved gene expression differences was maximised and proximate responses to diets minimised. The mRNA-seq results for each diet regime and body part were highly repeatable across independent biological replicates. qRT-PCR provided strong support for the genome-wide patterns of gene expression in the mRNA-seq. 214 transcripts showed DE above 2 log_2_OFC between treatments. Within this set, 94 transcripts were matched to homologous genes in *D. melanogaster* (44%). The functional description of these homologues showed evidence for divergence in gene expression between sexually mature adult males from both nutritional regimes.

The larval diets which defined the ASG and Starch regimes differed in caloric value and nutritional content, giving insight into the likely selection pressures to which each population was challenged. The ASG larval diet contained over twice the Kcal/L in comparison to the Starch. Also, the corn meal in the ASG diet offered additional sources of carbohydrates, proteins, and other dietary nutrients (http://ndb.nal.usda.gov/). It is this type of dietary complexity, rather than the caloric content *per se*, that we aimed to impose in our selection lines, as it is known to affect life history traits such as lifespan^78^. The successful effects of selection arising from exposure to these diets is evidenced by our previous report of dietary adaptation in male body mass at sexual maturity^53^.

The divergent nutritional selection during development resulted in a pattern of evolved differences in gene expression in adult males. Given the major differences between the diets in the complexity and diversity of carbohydrate content, DE in some form of nutrient metabolism was predicted. Indeed, homologues of DE genes in the abdomen tissues of males were functionally enriched for ‘Serine-type endopeptidase activity’. The finding that different suites of these peptidases, (putative digestive enzymes^79^) showed differential expression could suggest that differentiation in digestive strategies might contribute to the observed diet-associated divergence^53^. Several Drosophilid systems that exhibit divergence driven by host specialisation show similar, although more extensive, patterns of DE in nutrient related metabolic genes^9,31,80–82^. This effect is predicted on the basis that nutrients available in the larval diet can have large effects on survivorship and also have the potential to drive selection for optimal ability to utilise novel host nutrients. Within the medfly, the impact of larval rearing diet on male adult life history is substantial^83,84^. DE in metabolic genes could be associated with phenotypes manifested through effects on body size and nutrient reserves^85,86^, but also through the potential for metabolic programming of adult metabolic traits by larval conditions^87,88^.

Interestingly, divergent DE was observed in genes associated with OXPHOS in the Starch regime flies. Such genes are expressed in, or interact with, the mitochondria, which as centres of energy production with rapidly evolving independent genomes, are thought essential in adaptation and speciation^89,90^. For example, in the lake whitefish species complex (*Coregonus* spp.), divergence in OXPHOS gene expression in adult fish shows a tight relationship with the energetic phenotypes represented by two reproductively isolated morphs^91–97^. The tissue specific DE seen in OXPHOS genes between males from ASG and Starch backgrounds may be associated with the previously observed behavioural divergence (courtship activity) in these lines^53^. Starch males showed elevated levels of courtship behaviours related to the expression of pheromones^53^ and the up regulation of energy-related genes in Starch male abdomens could reflect the energetically costly process of pheromone biosynthesis^98,99^. The capture of signatures of change to OXPHOS gene expression is interesting because it has been suggested to be important in responses to novel diets through detoxification and altered patterns of host related hormone regulation^80,81^.

Olfactory and gustatory receptor (OGR) genes are often associated with population divergence by host specialisation^9,81,100^ and the establishment of prezygotic barriers during speciation^101^. Such genes mediate chemosensory responses and are associated with host recognition, but also perception of pheromonal communication^102^. DE was observed in this study in an OGR gene (OR67a-like), as well as two olfactory binding proteins (OBPs) (Obp19d, Obp99a). Patterns of DE in similar OBPs are seen between allopatric populations of *D. mojavensis* adapted to different host cacti and, in conjunction with other OBPs and behavioural genes, have been suggested to contribute to further population divergence^9^. Future improvements to the quality of the medfly genome will deepen our understanding further. However, our transcriptomic data presented here are important in giving key initial genomic and functional insights into dietary adaptation in this important and significant pest species.

Despite the previously reported nutritional adaptation^53^, and the evolved differences in gene expression described above, we found no evidence for evolved differences in diet-associated mating patterns. Hence, in principle the two larval rearing diets provided sufficiently distinct selective environments to drive divergence^53^ and resulted in gene expression differences in classes of genes such as proteases, OXYPHOS-related and odorant genes that are reported to be associated with divergence and adaptation in other species^9,31,81,82,91–97,103^. However, this did not result in a consistent signal of mating divergence between populations. This absence of mating isolation is consistent with the reported lack of significant RI across a global scale in this species^52,104^, despite the presence of significant behavioural differences in courtship song and behaviour^43,48^. Further differentiation has been observed between global populations in other life history traits such as growth rate, longevity, and sexual maturation^44,46^, as well as pre-adult traits^105^, and resilience to domestication (a potential proxy for an enforced change of host)^45^. The variance seen across these traits in global populations of medfly reflects overall genetic diversity of these populations, which is closely linked to the medfly’s invasion history^41,49^. Lack of divergence (isolation) between populations that vary globally is likely due to the effects of gene flow^42,49,106^, potentially mediated by invasions due to human transport^107^. This migration between populations has likely suppressed the capacity for specialisation, even though, as we show here it is potentially possible at the transcriptional level, and has instead maintained the plasticity exhibited by the medfly as a generalist^33^. Despite this, this study demonstrates that a laboratory population (over 20 years isolated from the wild) maintained sufficient genetic variation to respond to experimentally-imposed divergent selection. Relating this to natural populations, as quarantine measures become increasingly effective and reduce gene flow between global medfly populations^49^, suggests that the adaptive potential observed here may lead global populations to diverge further.

## Supplementary information

Supplementary Tables S1-S12

Supplementary Figures S1-S17

## Data Availability

Data are available on the GEO database^108^: GSE86029; GSM2291180 to GSM2291191. Raw data for the mating tests are included in Table S5.
