# Supplementary material for: Mate choice and gene expression signatures associated with nutritional adaptation in the medfly (Ceratitis capitata)
Source: Sci Rep. 2019 Apr 30;9:6704. doi: 10.1038/s41598-019-42610-2 (PMC6491435; doi:10.1038/s41598-019-42610-2)

Mate choice and gene expression signatures associated with nutritional adaptation in the medfly (*Ceratitis capitata)*

Will Nash[1,2], Irina Mohorianu[1,3†], Tracey Chapman[1†*]

[1]School of Biological Sciences, University of East Anglia, Norwich Research Park, Norwich, NR4 7TJ, UK.
[2]Earlham Institute, Norwich Research Park, Norwich, NR4 7UZ, UK.
[3]School of Computing Sciences, University of East Anglia, Norwich Research Park, Norwich, NR4 7TJ, UK.

† Co-corresponding authors, *E-mail: tracey.chapman@uea.ac.uk; i.mohorianu@gmail.com

Correspondence and requests for materials should be addressed to T.C. (email: tracey.chapman@uea.ac.uk)

Supplementary Information

Figures S1-S17

Figure S1

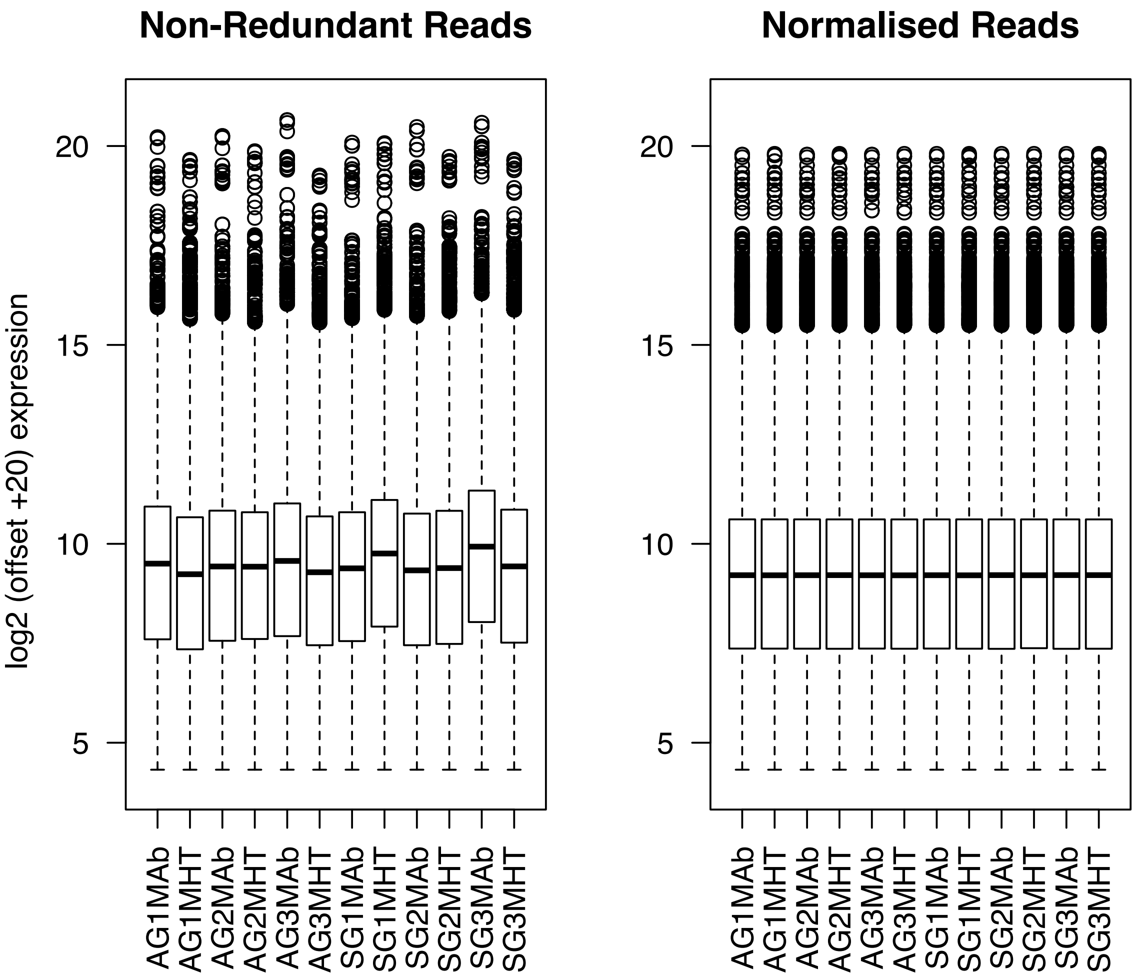

Figure S1. Distribution of expression levels before and after normalisation. Left panel represents the raw non-redundant reads, right panel represents the expression levels generated following the application of subsampling normalisation followed by quantile correction. On the X axis are the samples and, on the Y, the log2 expression levels with an offset of +20.

Figure S2

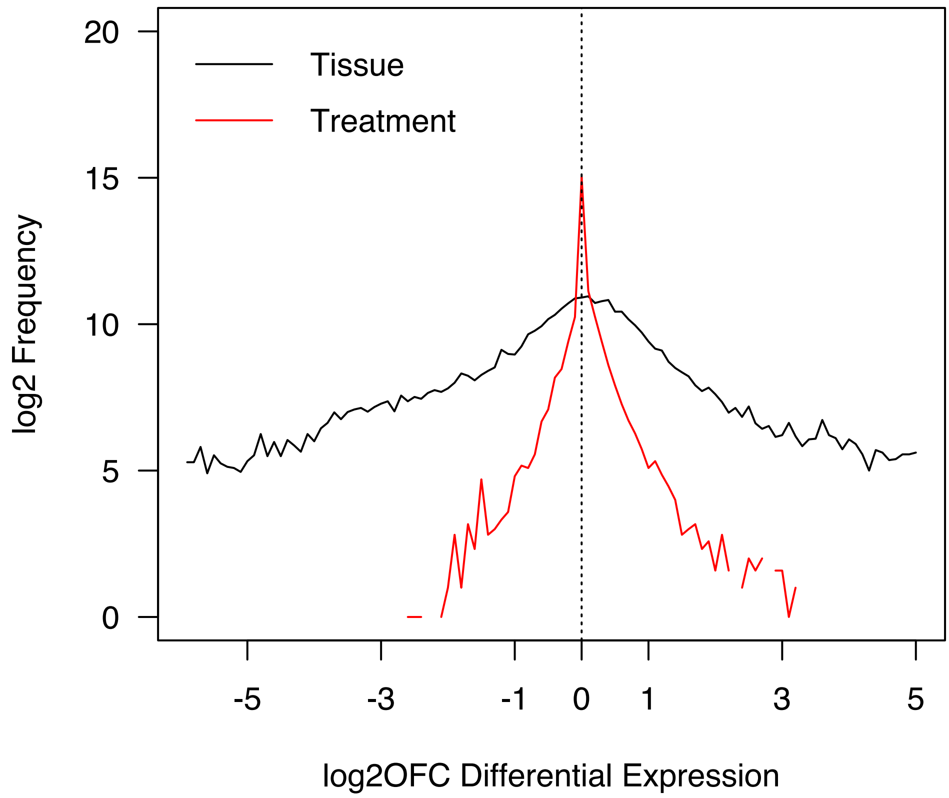

Figure S2. Hierarchical differential expression (DE) histogram. The frequency distribution of log2OFC DE between sum expression in tissue types (HT/Ab), and in treatment (A/S). The distribution of DE between treatment types, in red, falls below the distribution of DE between tissue types, in black.

Figure S3 gi|577721890

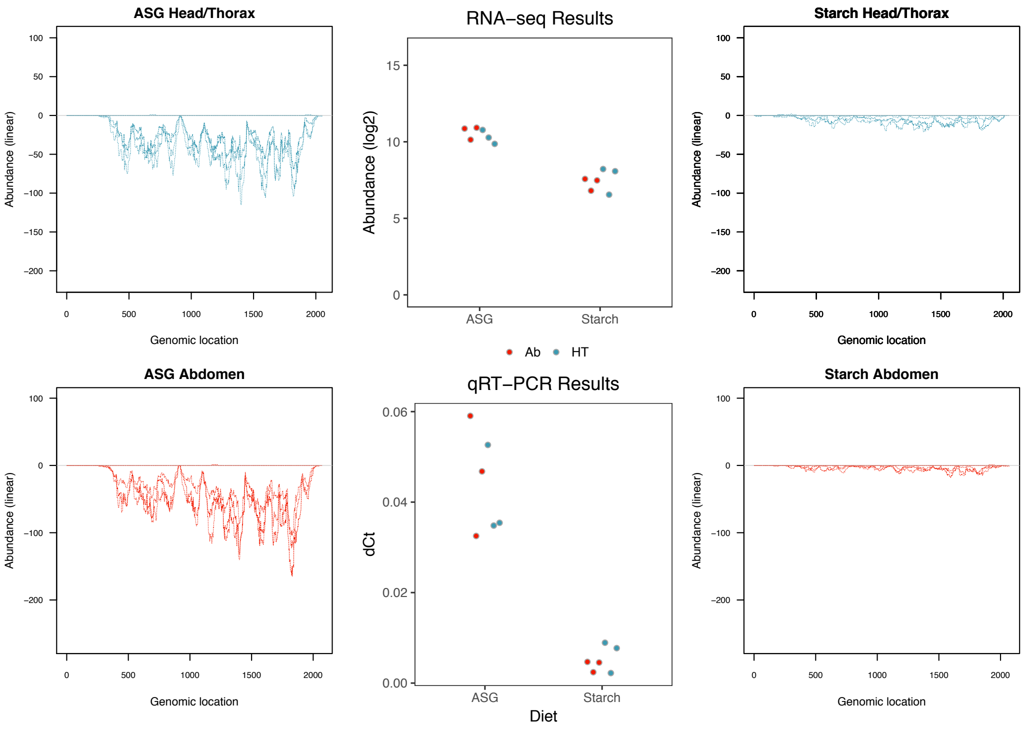

Figures S3 - S7. Validation on RNA-seq analysis using RT-qPCR for genes expresses in both HT and Ab Tissue. Each plot consists of a panel of six figures. The left- and right-hand columns are expression profiles of the candidate gene of interest (GOI). These expression profiles show the algebraic sum of abundances of incident reads at every position of the reference transcript, for each treatment. Expression levels from ASG tissue is presented in the left-hand column, expression levels from the Starch tissue in the right-hand column. Head /Thorax (HT) tissue is presented in the top row of both columns, and in blue. Abdominal (Ab) is presented in the bottom row of both columns, and in red. Line replicates are indicated by different line types. The central column of two figures shows the normalised expression level following analysis (top), and the $2^{-\Delta CT}$ expression level suggested by low throughput validation using RT-qPCR. Again, ASG left, Starch right, HT blue, Ab red.

Figure S4 gi|577728864

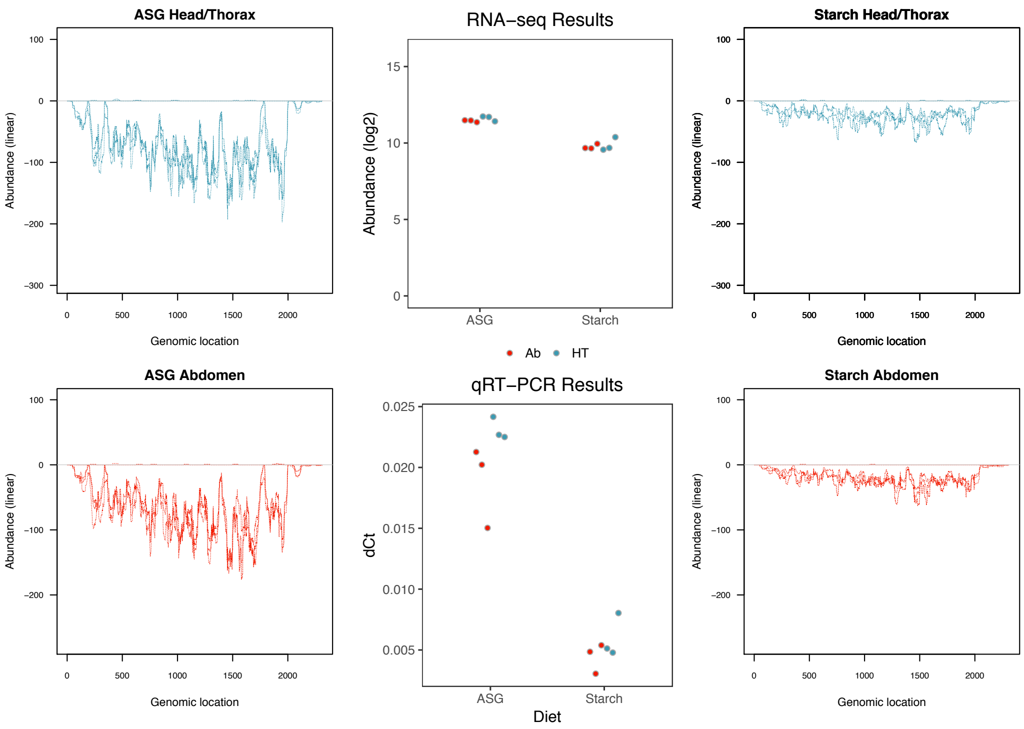

Figure S5 gi|577732748

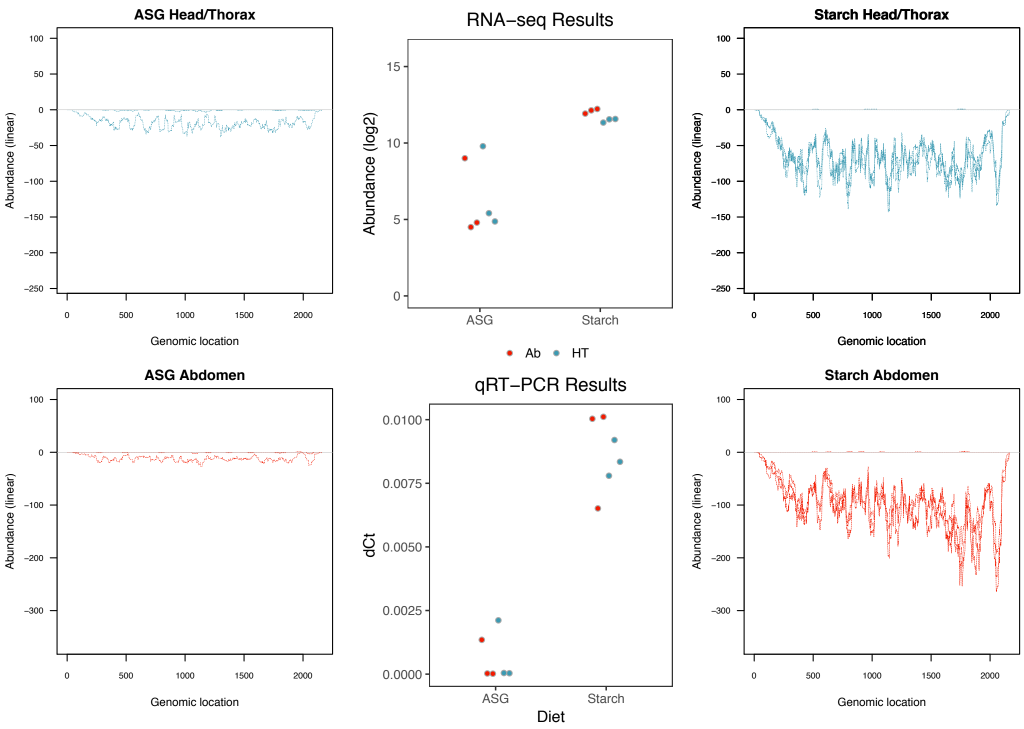

Figure S6 gi|577741672

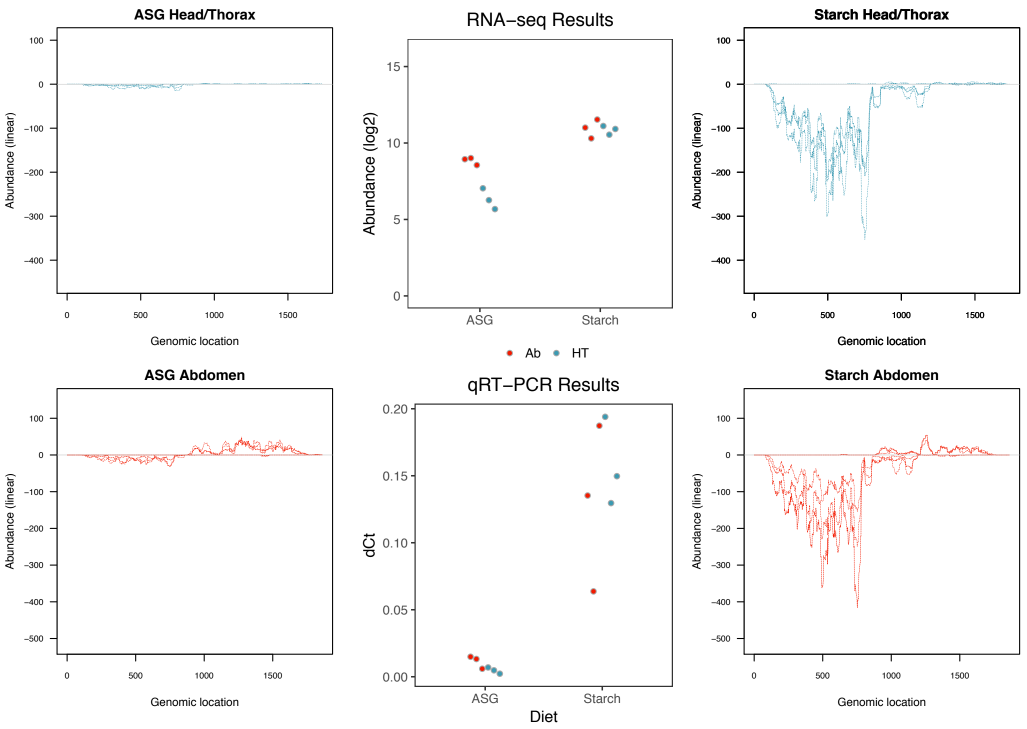

Figure S7 gi|577742368

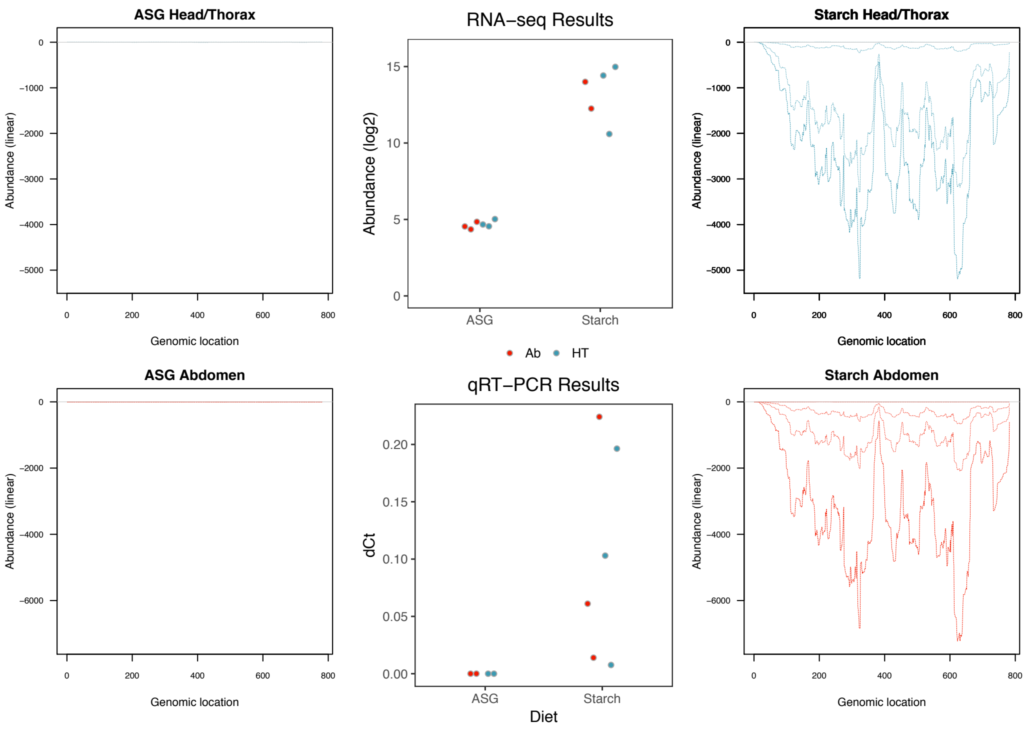

Figure S8 gi|577713500

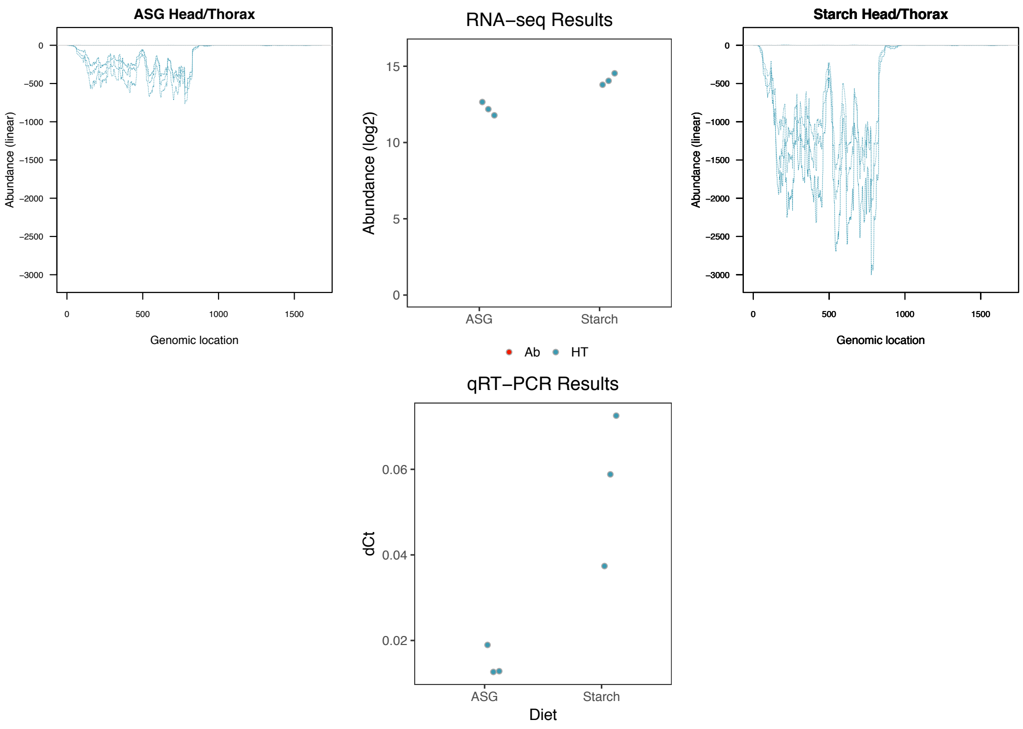

Figures S8 - S12. Validation on RNA-seq analysis using RT-qPCR for genes expresses in HT tissue only**.** Layout of these plots is as described for Fig. S3 – S7.

Figure S9 gi|577715176

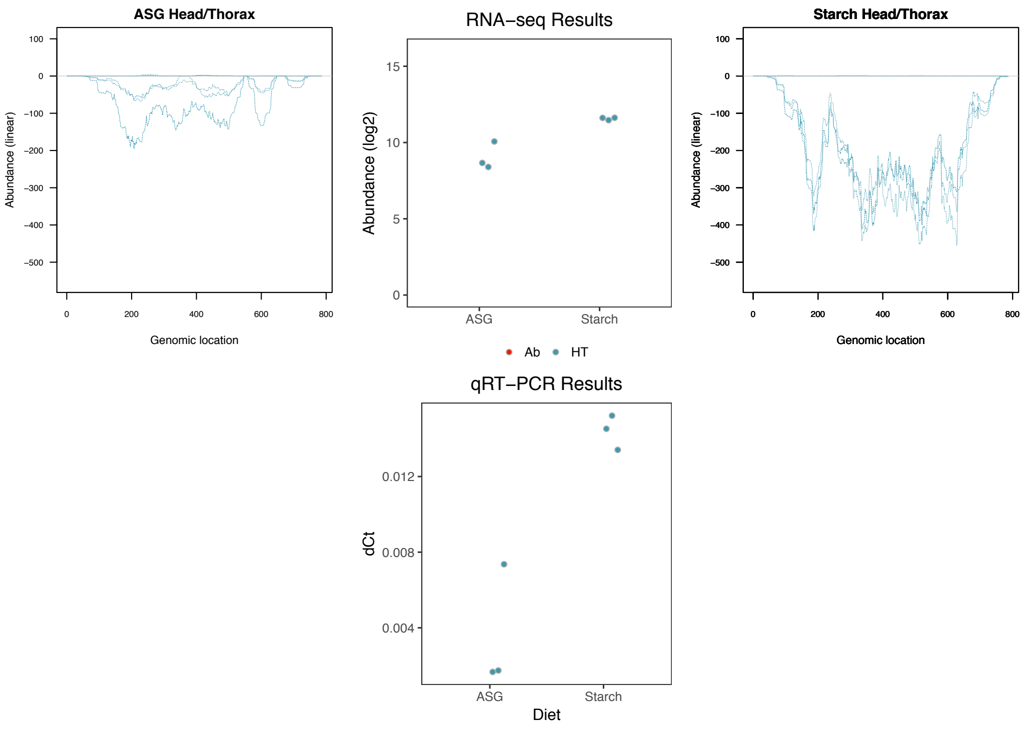

Figure S10 gi|577731920

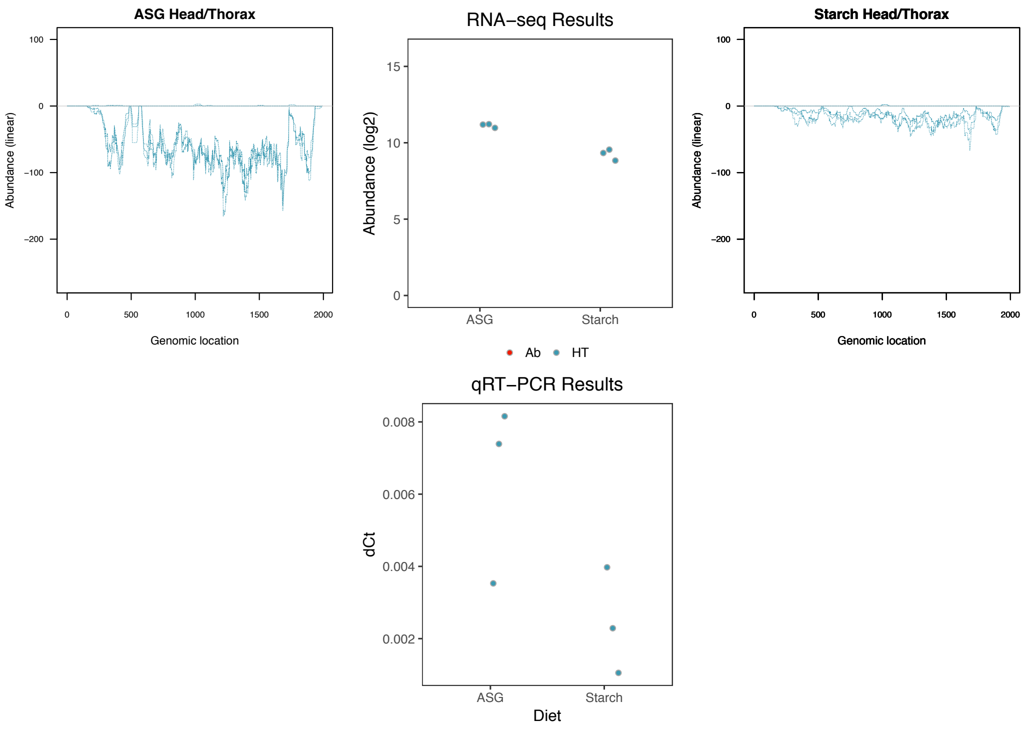

Figure S11 gi|577749646

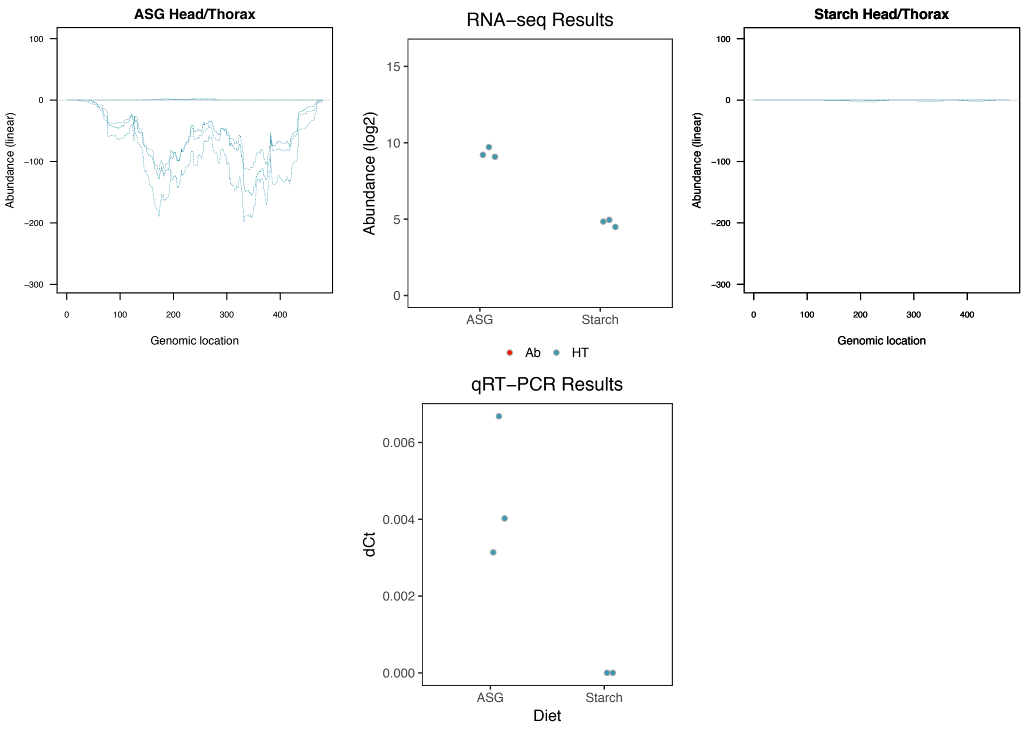

Figure S12 gi|807041305

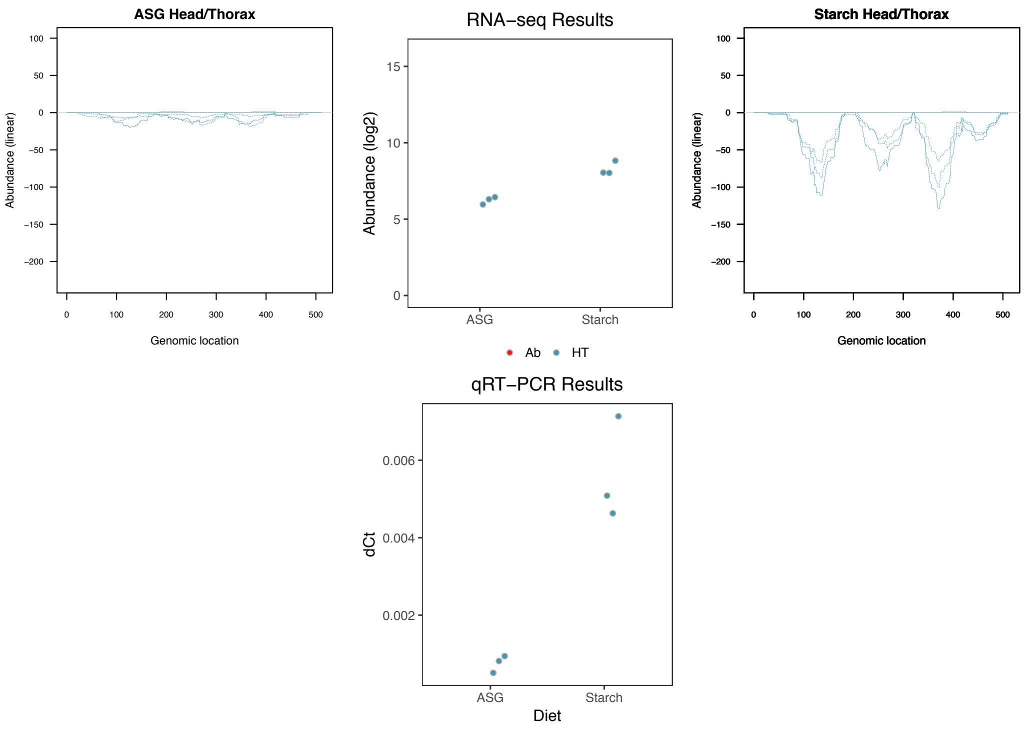

Figure S13 gi|577722828

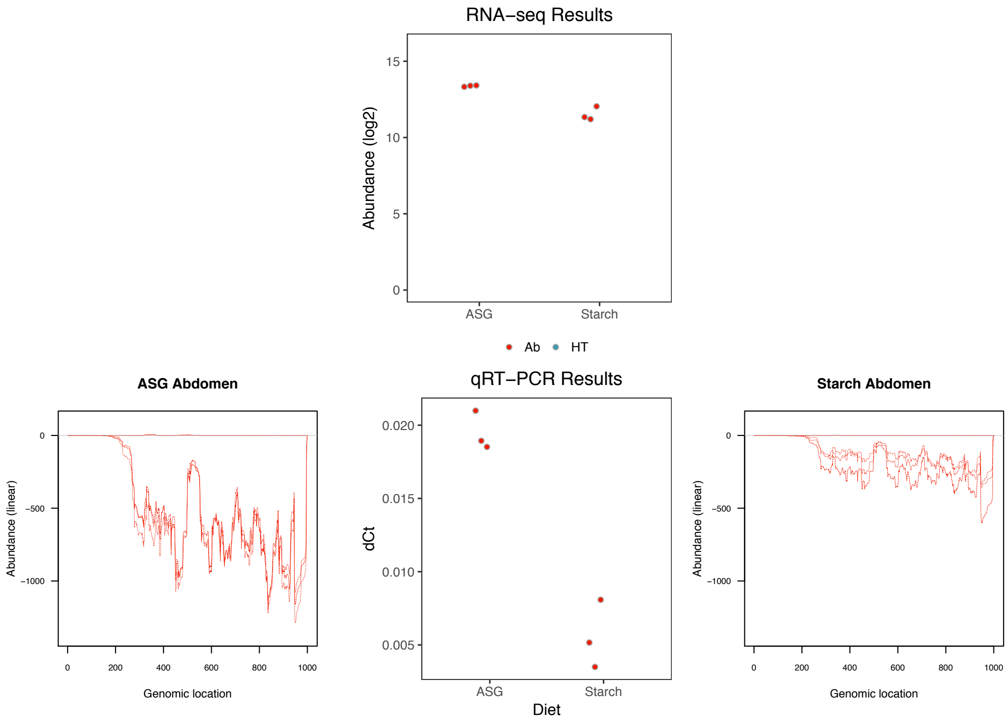

Figures S13 - S17. Validation on RNA-seq analysis using RT-qPCR for genes expresses in Ab tissue only**.** Layout of these plots is as described for Fig. S3 – S7.

Figure S14 gi|577742794

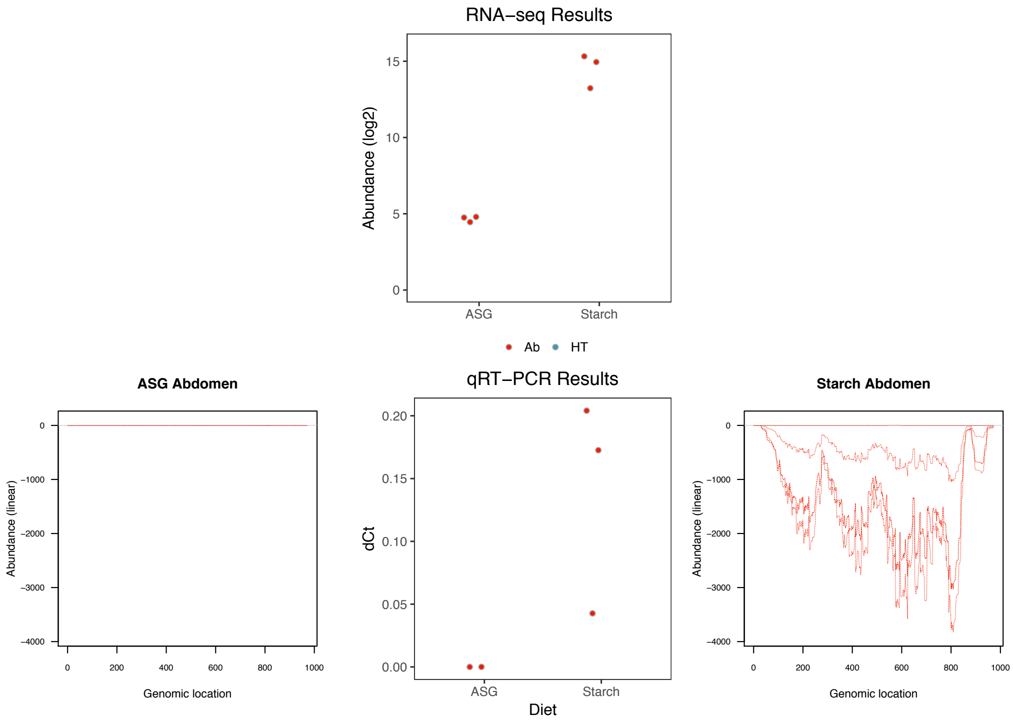

Figure S15 gi|577746780

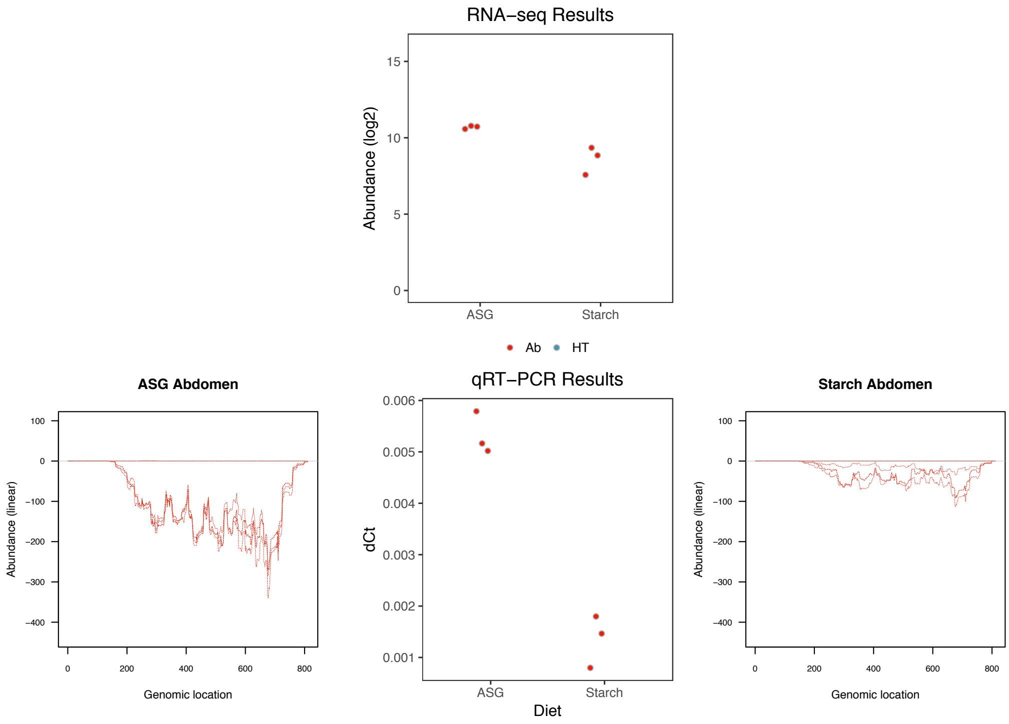

Figure S16 gi|807020514

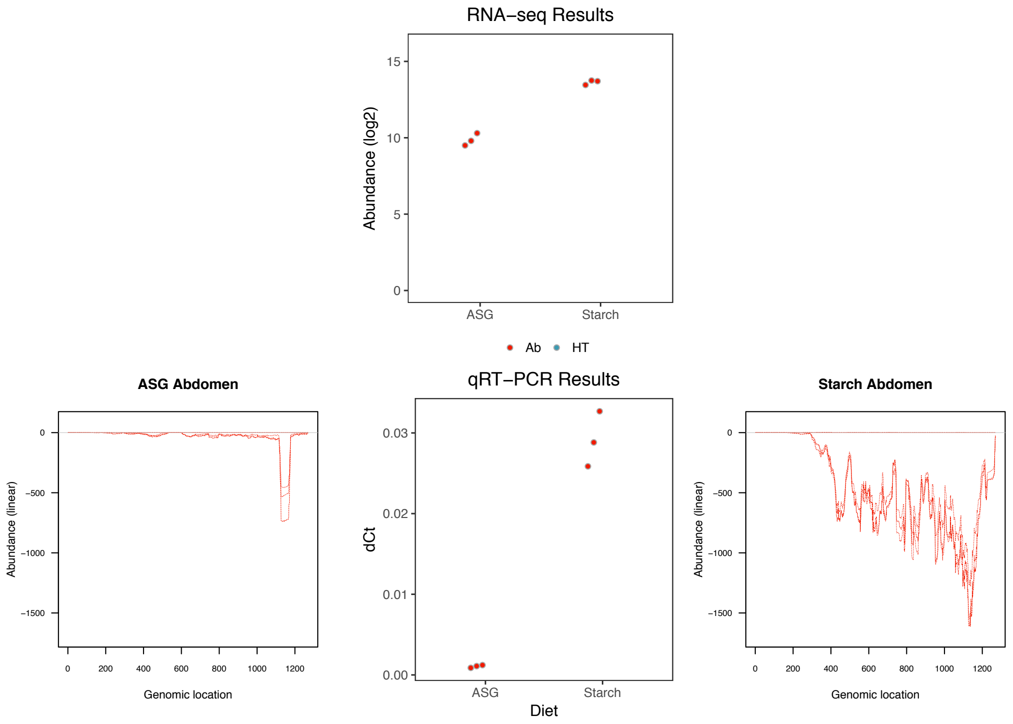

Figure S17 gi|89277021

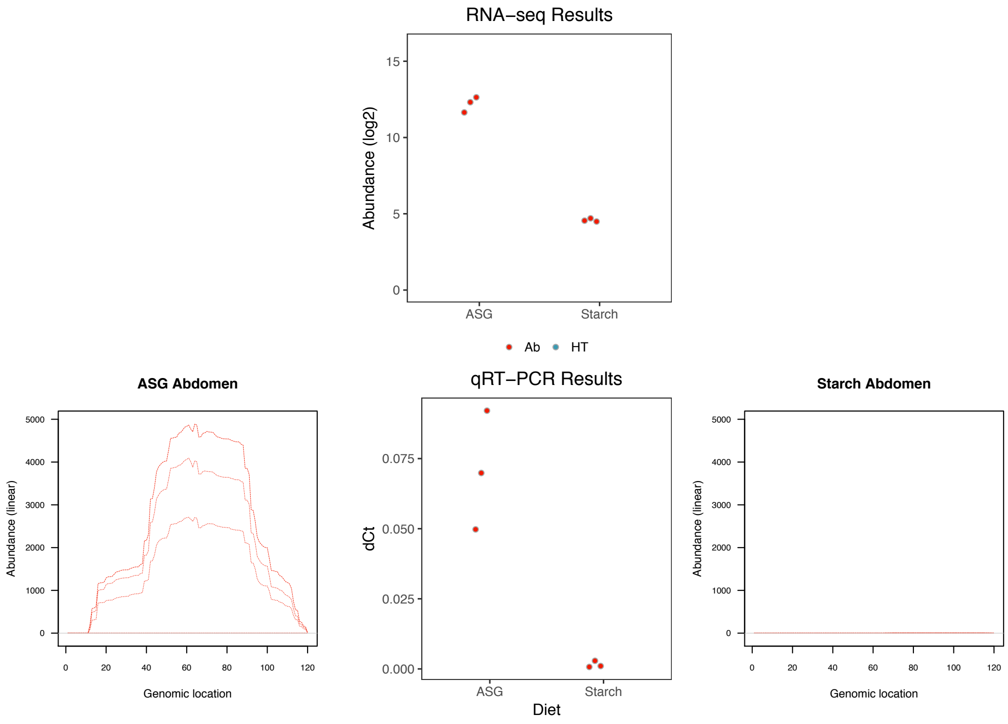

Supplement: Supplementary file 2 — Supplementary Figures S1-S17 [file 41598_2019_42610_MOESM2_ESM.pdf]
